# Understanding cultural significance, the edible mushrooms case

**DOI:** 10.1186/1746-4269-3-4

**Published:** 2007-01-11

**Authors:** Roberto Garibay-Orijel, Javier Caballero, Arturo Estrada-Torres, Joaquín Cifuentes

**Affiliations:** 1 Facultad de Ciencias, Universidad Nacional Autónoma de México, Apdo. Postal 113–100, Rumania N°700 Col. Portales, C.P. 03301, D.F, México; 2 Jardín Botánico, Instituto de Biología, Universidad Nacional Autónoma de México, Apdo. Postal 70–614, C.P. 04510, Ciudad Universitaria, D.F, México; 3 Laboratorio de Sistemática, Centro de Investigaciones en Ciencias Biológicas, Universidad Autónoma de Tlaxcala, Apdo. Postal 183, C.P. 90000, Tlaxcala, México; 4 Sección de Micología, Herbario FCME, Facultad de Ciencias, Universidad Nacional Autónoma de México, Apdo. Postal 70–181, C.P. 04510, Ciudad Universitaria, D.F, México

## Abstract

**Background:**

Cultural significance is a keystone in quantitative ethnobiology, which offers the possibility to make inferences about traditional nomenclature systems, use, appropriation and valuing of natural resources. In the present work, using as model the traditional mycological knowledge of Zapotecs from Oaxaca, Mexico, we analyze the cultural significance of wild edible resources.

**Methods:**

In 2003 we applied 95 questionnaires to a random sample of informants. With this data we integrated the Edible Mushroom Cultural Significance Index. This index included eight variables: frequency of mention, perceived abundance, use frequency, taste, multifunctional food use, knowledge transmission, health and economy. Data were analyzed in an inductive perspective using ordination and grouping techniques to reveal the behavior of species in a cultural multivariate dimension.

**Results:**

In each variable the species had different conducts. *Cantharellus cibarius s.l*. was the species with most frequency of mention. *Pleurotus *sp. had the highest perceived abundance. *C*.* cibarius s.l*. was the most frequently consumed species. *Gomphus clavatus *was the most palatable species and also ranked highest in the multifunctional food index. *Cortinarius *secc.*Malacii *sp. had the highest traditional importance. Only *Tricholoma magnivelare *was identified as a health enhancer. It also had the most economic importance. According to the compound index, *C. cibarius s.l.*, the *Amanita caesarea *complex, *Ramaria *spp. and *Neolentinus lepideus *were the mushrooms with highest cultural significance. Multivariate analysis showed that interviewees identify three main groups of mushrooms: species with high traditional values, frequent consumption and known by the majority; species that are less known, infrequently consumed and without salient characteristics; and species with low traditional values, with high economic value and health enhancers.

**Conclusion:**

The compound index divided the cultural significance into several cultural domains and showed the causes that underlie this phenomenon. This approach can be used in cross-cultural studies because it brings a list with the relative position of species among a cultural significance gradient. This list is suitable for comparisons and also it is flexible because cultural variables can be included or removed to adjust it to the nature of the different cultures or resources under study.

## Background

The Cultural significance (CS) of an organism has been defined as the importance of the role that the organism plays within a particular culture [1]. It has been used in ethnobotanical research in lexical retention [2,3]; to predict changes in the content of folk biological classifications, to asses the significance of a class of resource on the basis of its nomenclatural elaboration [1]; historical and archeological studies of human ecology and subsistence strategies [4-6]; perceptual salience of organisms [7]; and the borrowing of folk names, products and information about plants between cultures [8].

In earlier research, the CS of plant resources was estimated by simple scales of significance subjectively assigned by the researcher [*c.f*. [2,3,9]], but as Turner [8] points out these scales "are too simplistic to account for all the variables involved and not rigorous enough to be used with minimal bias". Furthermore these scales are restricted to the nature of the culture that is being studied, are established by the objectives of the researcher and do not allow cross-cultural analysis [1]. Hunn suggests that plant CS (practical significance in his terms) first must be described in sufficient detail to discriminate taxa from each other, and only then it can be measured. He also proposes that this description, known as activity signature, must be done from an intracultural or native perspective [ex. [10]].

Turner [8] developed the first theoretical model of CS. Her principal assumptions were that: CS is equal to use, when "use" is interpreted in its most general context, which means that knowing something is using it; every recognized plant have some degree of CS; and, CS vary in quality, intensity and exclusivity. The product of these three variables determines the "use value" of each use. Thus, her Index of Cultural Significance (ICS) of a plant is the sum of its "use values". However, these data are subjectively determined by the researcher [11] and not by informants in independent interviews. This model was modified by Stoffle et al. [12,13] based on the same assumptions, but adding the parts of a plant used for each purpose in the 'quality of use' category, and the 'contemporary use' variable category into the formula. More recently [14] also modified the Turner's model; they limited the answers categories for each variable to a binary system to make responses more objective; and they added a correction factor to the formula that modifies every use value with a measure of informant consensus. All previous techniques are concerned with measuring the CS so they include few variables where the importance of a resource is reflected, instead of using a mayor number of variables determining the CS.

Phillips and Gentry [15] proposed another way to measure the relative usefulness of plants, and refer to it as 'use value'. This was explicitly designed to allow hypothesis testing based on interviewing techniques, nature of data and statistics. The use value of a plant for an informant (UV_is_) is the average of the number of different uses assigned to that plant in several different interviews. The overall use value of a plant (UV_s_) is the average of the UV_is _of each informant. Phillips [11] classified this technique as part of the "informant consensus" methods that allows quantitative analysis of informants' knowledge. This approach, first proposed by Trotter and Logan [16] and Romney et al. [17], measures the relative importance of uses or species directly from the degree of consensus in the answers of informants in independent interviews [11]. Although informant consensus is efficiently used in ethno-pharmacological prospective surveys [16,18-20], it does not permit a thorough examination of the complex phenomenon of CS [21].

Pieroni [21] applied a compound index to edible plants, the Cultural Food Significance Index (CFSI). His index differs from earlier proposals because it is the first explicitly developed for food resources, and because it includes a more detailed group of factors influencing CS that will be treated in detail in methods.

Almost all efforts to evaluate the CS of resources have been focused on plants. Pieroni [21] was the first including in his dataset some (8) mushroom species, but his index does not take into account the particularities of mushrooms and the knowledge around them. Montoya et al. [22] used the frequency of mention from a free listing as an indicator of CS of mushrooms. By correlating these frequencies with the abundance and price of mushrooms, she found that the frequency of mention has a low but positive correlation with prices and a medium negative correlation with abundance. Although these two variables might be influencing the CS of mushrooms she proposed for further studies to take into account more variables (knowledge of habitats, fruiting season, morphology, recipes and eating preferences) to assess more precisely the cultural value of mushrooms.

**Figure 1:**
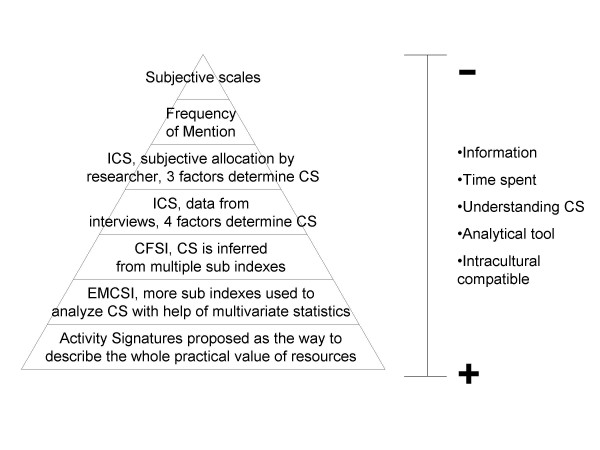
**Cultural significance study progress**. CFSI: Pieroni's Cultural Food Significance Index; CS: Cultural significance; EMCSI: Edible Mushrooms Cultural Significance Index; ICS: Turner's Index of Cultural Significance.

To recap, the study of CS of resources is a keystone in the development of an analytical and quantitative ethnobiology. It has many applications, but its successful use depends on the quality and accuracy of its measurements. That is why we have to understand it first and then measure it [1]. Through time, research has tended to give more detailed and complete descriptions of CS (Figure 1). However, compound indexes have to be thought of on the one hand, as tools to separate, analyze and understand the CS phenomena; and on the other, as techniques to estimate it.

In this paper, using the traditional mycological knowledge of Zapotecs from Oaxaca, as a model, we evaluate and analyze the CS of wild edible resources by a compound index. We measure the CS of edible mushrooms in function of their total score in a compound index; and undertake an inductive analysis of the reasons that determine the CS of edible mushrooms.

## Methods

### Study area

**Figure 2:**
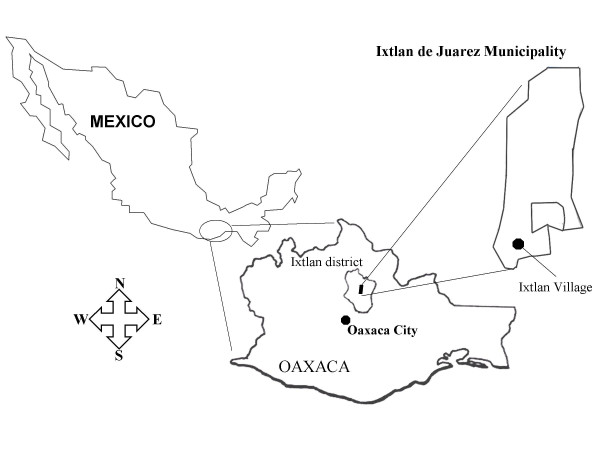
**Location of study area**.

**Figure 3:**
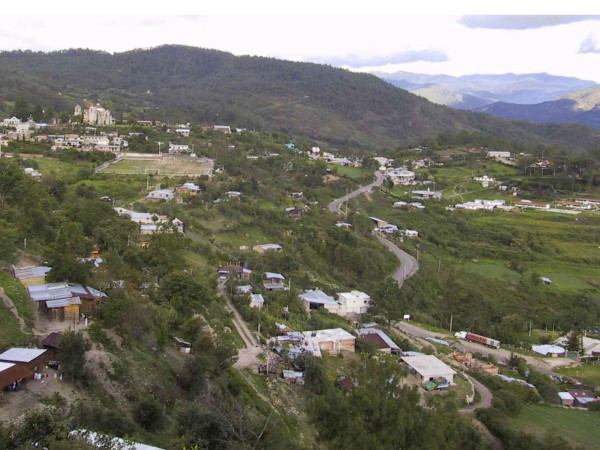
Ixtlan de Juarez village.

Ixtlan is located inside the Juarez ridge "Sierra de Juárez" in central Oaxaca, Mexico (Figure 2). For a complete description of its location, territory, climate and vegetation see Valdés et al. [23] and Garibay-Orijel et al. [24]. In general, it has temperate climate, and is located inside a wide and preserved coniferous woodland (Figure 3). In 2002 the village had approximately 2201 habitants with Zapotec origin but today just 50% speak their original language [25].

Zapotecs are an ancient culture that adopted Mayan Quiche cultural elements [26]. Monte Alban (650 – 900 B.C.) was its most representative pre-Columbian city, and after its abandonment their cultural unity became lost and fragmented [27]. Zapotec belongs to the otomague linguistic group, also containing mixtec, otomi, chinantec and mague. Nowadays they are widely distributed in Oaxaca, mainly in the Oaxaca valley, the Tehuantepec isthmus, the Juarez ridge, the Villa Alta district, Yalalag, and Miahuatlan ridge [27].

The economy in the Juarez ridge is based on agriculture, silviculture and cattling with some coffee and fruit plantations. Forest resources are very important to the region, with almost 40% of regional production based on them. In general, regional development is scarce, and health and education services are lacking [28]. Ixtlan is one of the most developed communities where approximately 60.43% of adult people are dedicated to primary activities (forest, agriculture and cattling); 9.02% work in schools; 9.02% in health and government offices; 12.72% in industry and 18.04% in services [25].

### Ethnomycological work

Since 2000 we have conducted an exhaustive recompilation of local traditional mycological knowledge. The taxonomy and nomenclature of folk taxa used in this paper are those documented in Garibay-Orijel et al. [24]. In May 2003, we applied 95 questionnaires to a random sample of informants. All informants were twenty years or older and they all lived at least for the last five years in Ixtlan. Fifty-one respondents were female and 44 male. Thirty-nine were between 20 and 39 years, 32 between 40 and 59 years, and 24 were 60 years old or more. Fifty-one were service employees, 18 were service employees and peasants, 12 were peasants, and 14 were forest employees. The questionnaire includes a free list and one question for every CS variable (sub index).

To obtain the free list [29], we asked informants to give us a list of every edible mushroom that they knew. We reviewed the correct taxonomical identity of every folk name given by each informant using high-resolution photographs (1200 dpi, 21.5 cm × 28 cm) as described by Garibay-Orijel et al. [24].

### Edible mushrooms cultural significance index

To develop the Edible Mushrooms Cultural Significance Index (EMCSI) we modified Pieroni's [21] model that includes seven cultural variables influencing CS: frequency of mention, perceived availability, frequency of use, taste score appreciation, plant parts used, multifunctional food use, and food-medicinal role. For EMCSI, we included from Pieroni's model the Mention Index (QI), Perceived Abundance Index (PAI), Frequency of Use Index (FUI), Taste Score Appreciation Index (TSAI) and Multifunctional Food Index (MFFI). Details of these variables can be found in Pieroni [21].

We eliminated Pieroni's Part Used Index because in plants, the roots, stem, leaves, flowers and fruits can be eaten alone or combined [12]. In Ixtlan in contrast, mushrooms are eaten as a whole and even if the stipe or cuticle are removed, there are hardly any cultural implications. It is important to mention that maybe in other places or cultures this variable could be useful and meaningful in terms of CS.

We eliminated also the Food-Medicinal Role Index because although in Mexico approximately 30 mushrooms (including lichens) are used with medicinal purposes [30], the food-medicine concept is not applied with them. This is, no mushrooms are consumed as nourishments and medicines at the same time. This contrasts with Asia (Korea, Japan, China) where this is quite common and almost 300 fungal species are used as medicines [31]. Instead of it we used the Health Index (HI). A very relevant factor influencing the CS of edible mushrooms is the possibility of becoming ill or dying after their consumption. Although plant toxicity is common too, people are always conscious that a mistake in mushroom identification could be fatal. HI evaluate where a species was placed by informants in the range between those species that are mislead because their toxicity or its similarity with toxic ones, and those that are eaten for health reasons.

In a general sense, "Culture" is defined as a socially patterned human thought and behavior with the properties of been shared, symbolic, integrated, learned, transmitted cross-generationally and adaptative [32]. From these characteristics, the last three are reflected in the appearance, permanence or extinction of resources uses; a matter not normally been part of CS evaluations. To assess this, we included the Knowledge Transmission Index (KTI).

Wild edible mushrooms are collected in more than 80 countries around the word; its sells estimated value is approximately $2 billion dollars a year. In rural areas, particularly in non-developed countries, the incomes due to mushrooms selling complete the economy of poor families [33]. For that reason, it could be expected that monetary value of mushrooms could affect substantially its CS in places where there are commercialized; we evaluated this with the Economic Index (EI).

### Calculation of each variable and final EMCSI compute

The final value of the Pieroni's index (CFSI) is the product its variables. Mathematical considerations in CFSI are: possible extreme values for each sub index are different (different scale); the possibility of zero values in must sub indexes is omitted, thus some characteristics may be overrated and the information of no CS is lost; the weight in the total calculation of each sub index is different.

To compute the compound index (EMCSI), we first categorized informants' responses to the questionnaire. Data for each variable were obtained as follows:

QI = (N°mentions/N°informants) 10.

**Figure 4:**
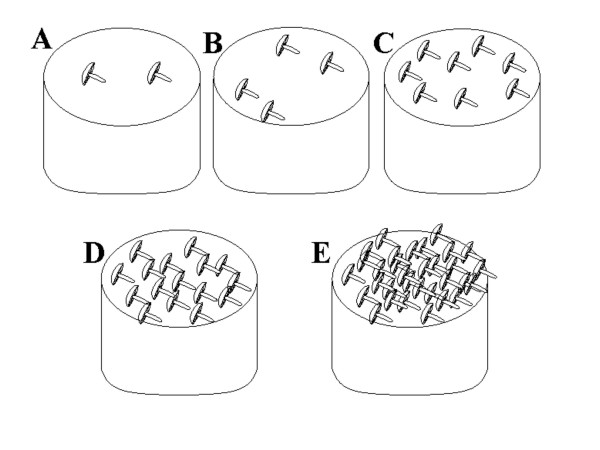
**Visual stimuli used in the perceived abundance test**.

PAI, informants rank the species perceived abundance based on a graphic stimulus that shows five possibilities on a logarithmic scale (Figure 4).

FUI, informants answer the options question: How often do you eat sp_i_?

**Figure 5:**
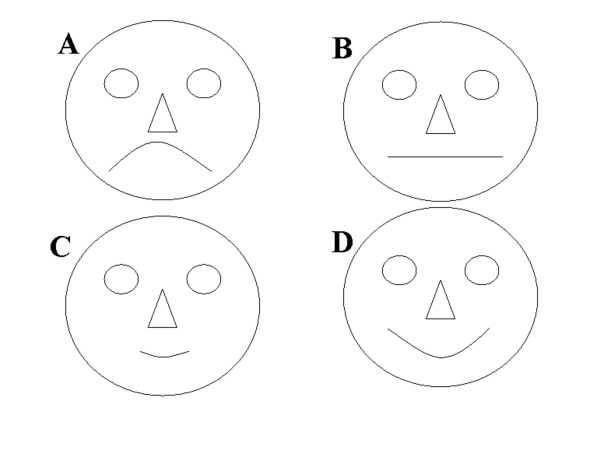
Visual stimuli used for the taste score appreciation test.

TSAI, informants answer the rank question: How much do you like sp_i_? To avoid the subjectivity of each informant, we used graphic stimuli to categorize their answers (Figure 5).

MFFI, informants answer the open question: How do you cook sp_i_?

KTI, we asked our informants how many generations were involved in the knowledge of certain mushroom. If it was a new use, we asked from whom they had learned it.

HI, informants answer the options question: How safe is to eat sp_i_, and, can its consumption be harmful? It is important to notice that the difference between HI and Pieroni's Food-Medicinal Role Index was done because our scale ranges from toxic to healthy foods and his scale ranges from healthy to medicinal foods.

EI, informants answered the option questions: Have you sold/bought sp_i _and at what price?

In EMCSI, all variables are based on a 0 to 10 scale and all indexes have the same weight. PAI, FUI, TSAI, MFFI, KTI, HI and EI are the average of all informants reporting a particular species. The relative value of mentions QI was used to amplify differences and to estimate the CS of species on the whole sample.

Table 1 shows the categorization of the possible responses to the open questions, the alternatives to the choice options, and the values in every variable for each answer.

**Table 1:** Answers categorization and values for the cultural significance sub Indexes

Sub index	Answer	Value
PAI	A	0
	B	2.5
	C	5
	D	7.5
	E	10
FUI	Never	0
	Not every year	2.5
	Every year once	5
	2–3 times a year	7.5
	4 or more a year	10
TSAI	A	0
	B	3.33
	C	6.67
	D	10
MFFI	Do not know	0
	Always mixed in a stew with other mushrooms and meat: "amarillito con carne y hongos"	2.5
	In a stew not as its principal element, mixed with mushrooms, not with meat: "amarillito con hongos"	5
	As the principal element of a stew: pie, "quesadillas", mushrooms soup	7.5
	Cooked alone not in stew: roast, fried in butter	9
	If it is eaten raw or conserved for future consumption	+1
KTI	New use, discovered by itself	0
	An immigrant (near town, other Mexican state, foreigner)	2.5
	Some town people, not blood parent (husband, friend, job partner)	5
	Father or mother, and he/she did not teach it to he's sons	7.5
	Three or more generations involved (grand fathers, fathers, he/she, sons)	10
HI	He/she do not eat it because can be confused with a toxic one	0
	He/she had eat it but with ill consequences	3.33
	He/she eat it with confidence, and it is healthy	6.67
	He/she eat it because it is good to health (give strong, mind power, reconstituent, medicine)	10
EI	He/she do not sell or buy it	0
	He/she have sell or buy it occasionally at low prices	3.33
	He/she have sell or buy it regularly	6.67
	He/she have sell or buy it at high prices	10

PAI: Perceived Abundance Index; FUI: Frequency of Use Index; TSAI: Taste Score

Appreciation Index; MFFI: Multifunctional Food Index; KTI: Knowledge Transmission Index; HI: Health

Index; EI: Economic Index.

The formula for the index was: EMCSI = (PAI+FUI+TSAI+MFFI+KTI+HI+EI)QI.

To clarify the procedures, in Table 2 we provide an example of a hypothetical questionnaire of one species for three interviewees, the categorization of answers and the compute process.

**Table 2:** Example of EMCSI compute process for the responses of three interviewees

Var.	Question	I 1	Cat.	Val.	I 2	Cat.	Val.	I 3	Val.	Compute
QI	Sp. mentioned in his/her free list	Yes		1	Yes		1	No	0	2/3 = 0.67*
PAI	Informant rank the abundance of sp_i_	B	B	2.5	C	C	5		0	3.75
FUI	How often do you eat sp_i_?	Every week	4 or more a year	10	Monthly from Jul. to Sep.	2–3 times a year	7.5		0	8.75
TSAI	How much do you like sp_i_?	D	D	10	C	C	6.67		0	8.335
MFFI	How do you cook sp_i_?	Fried in butter, raw	Cooked alone plus raw (9 + 1)	10	"Amarillito con hongos"	Not principal element	5		0	7.5
KTI	How many generations know...	Since grandma	Three or more generations	10	Learned from husband	Town, not blood parent	5		0	7.5
HI	How safe is to eat sp_i_...	Eat it with confidence		6.67	Because it is good to health		10		0	8.335
EI	Have you sold/bought sp_i_...	No		0	Buy every month	Regularly	6.67		0	3.335
EMCSI										**46.404**

In Var.: Variable. QI: Mention Index. PAI: Perceived Abundance Index. FUI: Frequency of Use Index. TSAI: Taste Score

Appreciation Index. MFFI: Multifunctional Food Index. KTI: Knowledge Transmission index. HI: Health Index. EI: Economic Index.

EMCSI: Edible Mushroom Cultural Significance Index. I 1, I 2, I 3: Answers of informants 1, 2 and 3. Cat.: Categorization. Val. Value. In the entire table, the categorizations and associated values are those indicated in Table 1. In Compute: * the formula for QI = (Number of mentions/Number of informants) 10. However, note than the division by 10 is to fit QI values to the same scale of other variables, because in this example just 3 informants were considered, the division was not done. The final value of each variable (except QI) is the average of informants' responses considering just those that knew the species; in this case 2.

### Analysis

As argued by Pieroni [21], indexes of CS could carry out more complex and comparative schemes when coupled with multivariate statistics; so in order to analyze relationships between species and sub indexes we developed a set of grouping and ordination techniques. First, with the species-by-sub index matrix, we calculated the Euclidean distances between species. Then we searched for groups of species with the complete linkage amalgamation rule. Second, to identify groups of species based on their similarity [34], we ran a multi-dimensional scaling analysis (MDS) with the Euclidean distances. We inferred the variables that arranged these groups with a Principal Component Analysis (PCA) by variables (columns). To explain the way each sub index is acting on the entire Cultural Significance process, we developed a PCA by OTUS (rows). We also looked for correlations between sub indexes with Spearman correlations [35]. Statistical procedures were performed using STATISTICA 5.1 for Windows [36] and BIODIVERSITY PRO 2 [37].

## Results and discussion

**Figure 6:**
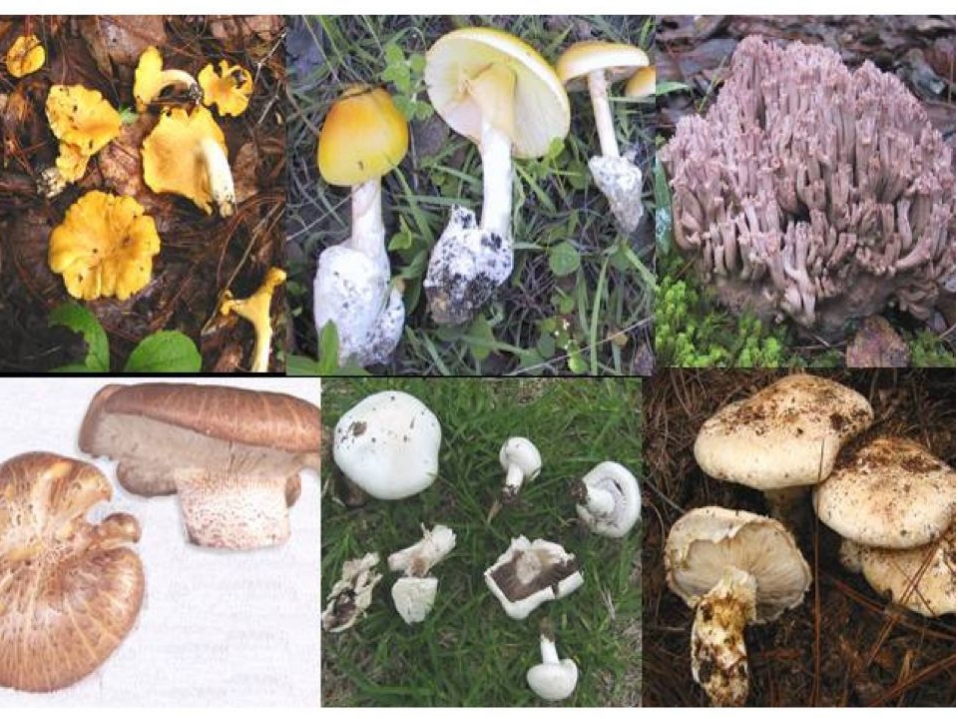
**Mushroom species with most cultural significance in Ixtlan**. Top, from left to right: *Cantharellus cibarius s.l., Amanita caesarea s.l., Ramaria purpurissima *(one of the many *Ramaria *species used in Ixtlan). Bottom, from left to right: *Neolentinus lepideus*, *Agaricus pampeanus*, *Tricholoma magnivelare*.

Twenty-one traditional taxa were mentioned in all free lists, which correspond to 37 scientific taxa (Table 3) [24]. *Amanita caesarea *complex, *Ramaria *spp., *Neolentinus lepideus *and *Agaricus pampeanus *were recognized by more than 50% of informants (Figure 6). If we group the two *Cantharellus cibarius *taxa, commonly considered as the single folk species "Beshia de", they had 89 mentions. *Tricholoma magnivelare*,*Hypomyces lactifluorum*,*Hydnum repandum s.l*. and *Lactarius volemus s.l*. were recognized between 50% and 20% of informants. Species known by less than five informants were *Austroboletus betula*, *Lactarius deliciosus s.l.*, *Laccaria vinaceobrunnea s.l.*, *Hygrophoropsis aurantiaca*, *Pleurotus *sp. and *Gomphus clavatus*. In Table 4 we show the values of every CS sub index for each taxa.

**Table 3:** Correspondence between scientific and folk taxa

Species	Folk species	Taxa as treated in this paper
*Agaricus pampeanus*	"Beshia sh que cuayo"	*Agaricus pampeanus*
*Amanita basii*	"Beshia bella"	*Amanita caesarea *complex
*A. jacksonii*	"Beshia bella"	*Amanita caesarea *complex
*A. laurae*	"Beshia bella"	*Amanita caesarea *complex
*A. tecomate*	"Beshia bella"	*Amanita caesarea *complex
*Austroboletus betula*		*Austroboletus betula*
*Cantharellus cibarius *sp.1	"Beshia de" de mercado	*Cantharellus cibarius *sp.1
*C. cibarius *sp.2	"Beshia de" de monte	*Cantharellus cibarius *sp.2
*C. cinnabarinus*	"Lo biinii"	*Cantharellus cinnabarinus*
*Cortinarius *secc. *Malacii *sp.	"Beshia be tzi"	*Cortinarius *secc. *Malacii *sp.
*Gomphus clavatus*		*Gomphus clavatus*
*Hydnum repandum *var.* album*	"Beshia beretze"	*Hydnum repandum s.l.*
*H. repandum *var.* repandum*	"Beshia beretze"	*Hydnum repandum s.l.*
*H. repandum *var.* rufescens*	"Beshia beretze"	*Hydnum repandum s.l.*
*H. umbilicatum*	"Beshia beretze"	*Hydnum repandum s.l.*
*Hydnum *sp.	"Beshia beretze"	*Hydnum repandum s.l.*
*Hygrophoropsis aurantiaca*	"Beshia de que ya yeri"	*Hygrophoropsis aurantiaca*
*Hygrophorus purpurascens*	"Beshia que biarida"	*Hygrophorus russula s.l.*
*H. russula*	"Beshia que biarida"	*Hygrophorus russula s.l.*
*Hypomyces lactifluorum*	"Beshia ya wela"	*Hypomyces lactifluorum*
*Laccaria amethystina*	"Beshia ladhi biinii"	*Laccaria vinaceobrunnea s.l.*
*L. bicolor*	"Beshia ladhi biinii"	*Laccaria vinaceobrunnea s.l.*
*L*. aff.* bicolor*	"Beshia ladhi biinii"	*Laccaria vinaceobrunnea s.l.*
*L. laccata *var.* pallidifolia*	"Beshia ladhi biinii"	*L. laccata *var.* pallidifolia*
*L. vinaceobrunnea*	"Beshia ladhi biinii"	*Laccaria vinaceobrunnea s.l.*
*Lactarius corrugis*	"Beshia ni tzi"	*Lactarius volemus s.l.*
*L. deliciosus*	Hongo de leche naranja	*Lactarius deliciosus s.l.*
*L. deliciosus *var.* deterrimus*	Hongo de leche naranja	*Lactarius deliciosus s.l.*
*L. volemus*	"Beshia ni tzi"	*Lactarius volemus s.l.*
*Neolentinus lepideus*	"Beyere"	*Neolentinus lepideus*
*Pleurotus *sp.		*Pleurotus *sp.
*Ramaria flava *var.* aurea*	"Beshia culirri"	*Ramaria *spp.
*R. purpurissima *var.* purpurissima*	"Beshia culirri"	*Ramaria *spp.
*R. rubricarnata *var.* verna*	"Beshia culirri"	*Ramaria *spp.
*R. cf. versatilis*	"Beshia culirri"	*Ramaria *spp.
*Sparassis crispa*	Cabeza de león	*Sparassis crispa*
*Tricholoma magnivelare*	Matzutake	*Tricholoma magnivelare*

In Folk species, quoted names are in Zapotec, the rest in Spanish.

**Table 4:** Sub indexes values and edible mushroom cultural significance index estimates

**N°**	**Taxa**	**QI**	**PAI**	**FUI**	**TSAI**	**MFFI**	**KTI**	**HI**	**EI**	**EMCSI**
1	*Agaricus pampeanus*	5.053	5.698	3.840	6.350	6.500	8.641	6.521	0.000	189.731
2	*Amanita caesarea *complex	9.263	5.255	5.703	8.667	8.616	8.435	6.235	0.210	399.430
3	*Austroboletus betula*	0.421	4.167	4.833	4.168	7.625	5.625	6.670	0.000	13.932
4	*Cantharellus cibarius *sp.1	4.840	8.429	6.143	8.537	6.325	8.598	6.751	3.641	234.450
5	*Cantharellus cibarius *sp.2	4.530	8.162	5.974	8.685	7.059	8.718	6.755	0.167	206.210
6	*Cantharellus cinnabarinus*	1.158	3.889	4.444	7.619	7.500	6.786	6.670	0.000	42.735
7	*Cortinarius *secc. *Malacii *sp.	1.474	4.231	3.654	6.429	5.833	9.464	5.955	0.000	52.414
8	*Gomphus clavatus*	0.105	2.500	5.000	10.000	9.000	5.000	6.670	0.000	4.018
9	*Hydnum repandum s.l.*	3.895	3.603	4.571	7.224	6.674	8.958	6.762	0.180	147.895
10	*Hygrophoropsis aurantiaca*	0.421	6.250	5.000	6.670	2.500	5.000	6.670	0.000	13.512
11	*Hygrophorus russula s.l.*	0.526	5.833	2.643	4.443	3.000	6.786	6.670	0.000	15.461
12	*Hypomyces lactifluorum*	4.526	3.472	3.525	6.667	6.540	8.654	6.670	0.079	161.171
13	*Laccaria vinaceobrunnea s.l.*	0.421	5.625	3.125	6.668	3.125	8.750	6.670	0.000	14.300
14	*Laccaria laccata *var. *pallidifolia*	1.053	7.500	2.700	4.165	4.063	6.750	6.670	0.000	33.524
15	*Lactarius deliciosus s.l.*	0.421	5.000	1.500	8.335	8.500	7.500	6.670	0.000	15.792
16	*Lactarius volemus s.l.*	4.105	2.786	4.255	6.609	6.888	7.715	6.697	0.222	144.386
17	*Neolentinus lepideus*	6.737	3.194	3.230	9.235	7.500	8.320	6.779	1.251	266.164
18	*Pleurotus *sp.	0.421	8.750	2.500	6.670	6.250	2.500	6.670	0.000	14.038
19	*Ramaria *spp.	8.211	6.162	4.770	6.713	6.331	8.377	6.714	0.084	321.439
20	*Sparassis crispa*	0.737	0.500	3.000	9.334	5.000	4.500	6.670	1.427	22.423
21	*Tricholoma magnivelare*	4.842	3.667	1.944	8.391	6.682	3.649	8.422	4.565	180.707

N°: Number of the species. QI: Mention Index. PAI: Perceived Abundance Index. FUI: Frequency of Use Index. TSAI: Taste Score

Appreciation Index. MFFI: Multifunctional Food Index. KTI: Knowledge Transmission index. HI: Health Index. EI: Economic Index.

EMCSI: Edible Mushroom Cultural Significance Index.

### Cultural variables (sub indexes)

#### Perceived abundance index

Species with perceived abundance values of 7.5 or more were *Cantharellus cibarius *sp.1, *C. cibarius *sp.2, *Pleurotus *sp. and *Laccaria laccata *var. *pallidifolia*; and those perceived as rare (PAI ≤ 2.5) were *Gomphus clavatus *and *Sparassis crispa*. Certainly *L. laccata *var. *pallidifolia *is the most abundant mushroom in Ixtlan woods [23]. Previous research [38] has shown that *C. cibarius *sp.2 scores as common, but not abundant. Perhaps this can be explained by the fact that people think it is abundant by association with *C. cibarius *sp.1 that is sold copiously in the village market. We do not have data about *Pleurotus *sp. abundance, however forest employees reported that it is scarce near town, and abundant in wet faraway forests. Our data shows that *G. clavatus *and *S. crispa *are very uncommon and restricted to particular habitats. Perceived abundance index is the only EMCSI sub index that is not eminently cultural, because it is derived from the perception of an ecological aspect. The relation between real abundance and perceived abundance is not clear and needs further research. A clear understanding of this is fundamental to know how people appreciate and use their natural resources.

#### Frequency of use index

Only *Cantharellus cibarius *sp.1, *C. cibarius *sp.2 and the *Amanita caesarea *complex were used more than one time a year (FUI > 5). Those used in occasional years (FUI ≤ 2.5) were *Pleurotus *sp., *Tricholoma magnivelare *and *Lactarius deliciosus s.l*. The formers are species much appreciated and easy to obtain (by collect or commerce). The latter are mushrooms with regular abundances known only by restricted social groups.

#### Taste score appreciation index

According to informants, fifteen species can be cataloged as good tasting (TSAI ≥ 6.67). The most palatable were *Gomphus clavatus*, *Sparassis crispa*, *Neolentinus lepideus*, *Cantharellus cibarius *sp.2, the *Amanita caesarea *complex and *C. cibarius *sp.1. Those with simple taste (3.33 < TSAI > 6.67) were *Austroboletus betula*, *Laccaria laccata *var. *pallidifolia*, *Hygrophorus russula s.l.*, *Agaricus pampeanus*, *Cortinarius *secc. *Malacii *sp. and *Lactarius volemus s.l*. No mushroom scored as bad tasting. Because personal evaluations of taste are strongly influenced by the idiosyncrasy [39], cultural domains as "good taste" are only explicable by intracultural perspectives. As an example, Ruán et al. [40,41] discuss that the high CS of *Schizophyllum commune *as food in the tropics, particularly in Southwest Mexico, is not affected by its corky or rubbery consistence. In Ixtlan, between those species highly valued by their taste, we found: worldwide fungal delicatessen's as *C. cibarius s.l*. and the *A. caesarea *complex; species valued by local people because its similar to meat consistency (*S. crispa*, *N. lepideus*), a very common phenomenon in Mexico; and *G. clavatus*, a mushroom without previous reports of edibility in Mexico. The taste of some species was defined as simple (*L. laccata *var. *pallidifolia*), not consistent (*A. betula*) or bitter *H. russula s.l*. The last example is interesting because this taxon in fact are two species *H. russula *and *H. purpurascens *that people recognize as one "Beshia biarida". People commonly reported on the bitterness of this mushroom, relating it to either age of mushroom, or to its cuticle. Both species are edible, although locally one of them has a bitter taste. The lack of deep local folk taxonomic detail affects the use of these resources by not being able to tell them apart.

#### Multifunctional food index

Six species were consumed as principal stew elements (MFFI ≥ 7.5) and those occasionally consumed on their own (MFFI ≥ 8.25) were *Gomphus clavatus*, the *Amanita caesarea *complex and *Lactarius deliciosus s.l*. Mushrooms which are always consumed mixed with other mushrooms and meat (2.5 < MFFI > 5) were *Hygrophoropsis aurantiaca*, *Hygrophorus russula s.l*. and *Laccaria *spp. A series of practical factors take place in the decision of how to cook fungi: how many mushrooms of each species are available; the economic status, since poorer people substitute meat with fungi; how much time they have to prepare mushrooms; and how hungry they are. Other factors are cultural and idiosyncratic: the culinary background of the culture, family traditions and recipes, and the individual tastes. *Gomphus clavatus *and *L. deliciosus s.l*. are consumed on their own in Ixtlan by the few people that know them and appreciate their flavor. The *Amanita caesarea *complex species are an interesting case, they are eaten alone by almost all the interviewees, because of flavor, size and easiness to be cooked. The extreme of this culinary value takes place inside the woods with forest employees. When they camp for several days, they complement their diet by eating wild fungi, and these mushrooms go from the ground to the campfire to the mouth quickly and pleasantly. On the other hand, species that are always mixed with other mushrooms and meat have several characteristics. They are very abundant, small and simple in flavor (*Laccaria laccata *var. *pallidifolia*); abundant and big but not very tasty (*Hygrophorus russula s.l.*); and common, small and simple tasting (*Hygrophoropsis aurantiaca*). There is a group of species (*Hydnum repandum s.l*., *Cantharellus *spp. and *Ramaria *spp.) commonly cooked mixed or alone in "amarillito", a kind of yellow chili sauce "mole" with ritual and festive implications. *Lactarius volemus s.l*. was reported to be consumed raw by two informants. *Cantharellus cibarius *spp., *L. volemus s.l*. and *Neolentinus lepideus *were dried up. This practice is more important in *N. lepideus *because: it is a rare mushroom, found by few people; commonly sold, given as gifts or bought as something special; specimens can reach almost 30 cm; and due to its phenology (April and May) it can only be enjoyed during a short period.

#### Knowledge transmission index

According the KTI, eighteen folk species are part of the mycological traditional knowledge of the people of Ixtlan (KTI ≥ 5). The most deep-rooted were *Cortinarius *secc. *Malacii *sp., *Hydnum repandum s.l.*, *Laccaria vinaceobrunnea s.l.*, *Cantharellus cibarius *sp.2, *Hypomyces lactifluorum*, *Agaricus pampeanus*, *C. cibarius *sp.1 and the *Amanita caesarea *complex. One can usually trace the knowledge of these mushrooms in up to six generations in one family, although there is a tendency of not teaching children the traditional knowledge about mushrooms. This is evident in *C*. secc.* Malacii *sp. that nowadays is known just by 14 informants of which only two were young people. This can be illustrated also with the case of *L. vinaceobrunnea s.l*. since although previous generations knew it well (KTI = 8.750), today only 4.21% of informants are familiar with it. Those mushrooms with less traditional importance (KTI < 5) were *Sparassis crispa*, *Tricholoma magnivelare *and *Pleurotus *sp. These data corroborate previous observations about the intense cultural exchange and incorporation of new species to the traditional mycological knowledge of the town [24]. In our observations, Ixtlan inhabitants apparently did not originally know *S. crispa*, but have learned to consume it from sellers from neighboring towns. In fact, *T. magnivelare *(American matsutake) is a recent (10–15 years ago) incorporation to Ixtlan' culture. Today Ixtlan' people use this mushroom with their own new ideas and myths in their cultural context. This evidence shows the relevance of this variable to understand the dynamic and adaptive nature of traditional knowledge.

#### Health index

*Tricholoma magnivelare *(HI > 8.336) was the only mushroom believed to have health enhancing properties, including "strength", "virility" and "intelligence". Other mushrooms were also believed to be health related by classifying them as "nutritious" or "good for the body", such as *Neolentinus lepideus*, *Hydnum repandum s.l.*, *Cantharellus cibarius *spp., *Ramaria *spp. and *Lactarius volemus s.l*. (HI > 6.670). Species avoided because of their resemblance to toxic ones (HI < 6.67) were *Agaricus pampeanus*, the *Amanita caesarea *complex and *Cortinarius *secc. *Malacii *sp. When people where questioned about the special health properties of *T. magnivelare*, in general they follow a similar logical path: "Japanese companies buy this mushroom at prices never seen for mushrooms"; so, "they have to extract something special from this mushroom"; "as Japanese are very smart and healthy"; "those extracts must be medicines or vitamins". In fact, some informants assured feeling healthier, or reported that their children get better grades in school since they eat them. Species considered as more than healthy, according informants, are "special", "very consistent" or have nutritional and metaphysical properties, such as "full with vitamins", "almost as medicine", "relaxing", "it fills the tummy", "is better than meat". Taxa avoided by certain people can be truly confused with toxic ones present in Ixtlan' woods. White *Agaricus *like *A. pampeanus *can be confused with *Amanita virosa *buttons [42]; in fact, one mortal intoxication in Ixtlan was due to this last species. Old or washed *A. muscaria *specimens could be mistaken for *A. caesarea s.l*. [43] and *Cortinarius *secc. *Malacii *sp. is inside a genus with very similar toxic species [44].

#### Economic index

Eleven folk taxa had zero EI values, these were species never sold in Ixtlan. *Hypomyces lactifluorum, Ramaria *spp., *Cantharellus cibarius *sp.2, *Hydnum repandum s.l.*, the *Amanita caesarea *complex and *Lactarius volemus s.l*. had 0 < IE ≤ 1 values. These were occasionally sold by other town sellers that offer mushrooms in Ixtlan on a door-to-door basis. Only four species had an appreciable economic importance (EI > 1), *Neolentinus lepideus *and *Sparassis crispa *had values between, 1 and 3.33. The former, is found occasionally by forest employees and people buy it from them at any price. The latter is a mushroom not common in Ixtlan woods, so it only can be bough from other town sellers. *Cantharellus cibarius *sp.1 is the sole mushroom regularly sold in Ixtlan' market (IE = 3.641). Every Monday from July until October its possible to buy 1/2 kg of these mushrooms from $1.5 to $2 USD (11.5 pesos/dollar). *Tricholoma magnivelare *had the highest EI value (IE = 4.565), although people do not sell it anymore to Japanese companies. This is very significant because it means that the economic importance is not tied only to income or expenses, it is also related to its potential economic value.

### Edible mushrooms cultural significance index

The EMCSI values varied from 399.430 for the *A. caesarea *complex to 4.018 for *G. clavatus*. Species with highest scores were the *A. caesarea *complex, *Ramaria *spp., *N. lepideus*, *C. cibarius *sp.1 and *C. cibarius *sp.2. The "Beshia de" folk taxa in conjunct reached 440.660 points, which is more than any other fungi. Species with less CS (EMCSI < 15) were *Laccaria vinaceobrunnea s.l., Pleurotus *sp., *A. betula*,* H. aurantiaca *and *G. clavatus*. Because EMCSI is pondered by the mentions relative value, it must be considered a sample CS estimate. Thus, species with more mentions count higher than those with fewer mentions.

Interestingly, Montoya et al. [45], although using a free listing technique, found that *Amanita caesarea s.l*. and *Cantharellus cibarius s.l*. are among the three most cultural significant mushrooms to the nahua communities inhabiting the Malinche National Park in Tlaxcala, center of Mexico. These two taxa are indeed very appreciated thru the world; however, if they are the most cultural important species in Mexico is an answer waiting for cross-cultural studies achievable by techniques as the proposed in this paper. Other important question to deal in the future is if data from a free list and from a compound index could be comparable and which of these techniques is better to deal with the CS phenomenon.

For mushrooms, other cultural domains to include in a compound index could be the parts used, its medicinal role, its religious or ritual use and several economic related issues like volumes of sell or collect, average prices, commercialization process, etc. Because the ethnomycologic study in Ixtlan has been done for several years, it is very likely that any of these variables affect locally the CS of mushrooms. In order to choose the variables that will be included in a compound CS index, it is imperative to have a previous scheme of the local traditional knowledge. A very interesting approach to do this accurately and time saving is to perform a preliminary inquiry about the causes of CS by the informant point of view. This is done by the question: what (mushroom, plant, etc.) is most important to you? Followed by: which criteria do you used to define the importance? By these way the own people is going to show the cultural domains locally relevant, and then their responses can be summarized into concrete cultural domains to include in the compound index [46].

### Multivariate analysis

**Figure 7:**
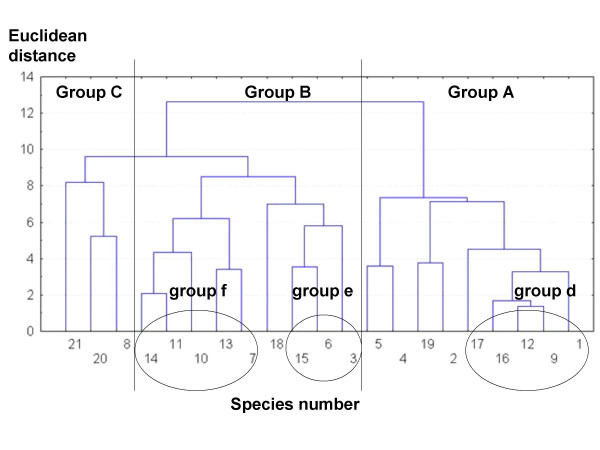
**Tree diagram for euclidean distances between species, groups formation by complete linkage**. Lines divide mayor mushroom groups designed by capital letters. Ellipses show minor mushroom groups designed by lowercase letters. 1: *Agaricus pampeanus*; 2: *Amanita caesarea *complex; 3: *Austroboletus betula*; 4: *Cantharellus cibarius *sp.1; 5: *Cantharellus cibarius *sp.2; 6: *Cantharellus cinnabarinus*; 7: *Cortinarius *secc. *Malacii *sp.; 8: *Gomphus clavatus*; 9: *Hydnum repandum s.l.*; 10: *Hygrophoropsis aurantiaca*; 11: *Hygrophorus russula s.l.*; 12: *Hypomyces lactifluorum*; 13: *Laccaria vinaceobrunnea s.l.*; 14: *Laccaria laccata *var. *pallidifolia*; 15: *Lactarius deliciosus s.l.*; 16: *Lactarius volemus s.l.*; 17: *Neolentinus lepideus*; 18: *Pleurotus *sp.; 19: *Ramaria *spp.; 20: *Sparassis crispa*; 21: *Tricholoma magnivelare*.

The tree diagram of Euclidean distances (ED) (Figure 7) showed three mayor groups of species. From right to left, the first group (A) is separated from the other groups at a ED of 12.62. It is conformed by the ten most culturally significant species except *Tricholoma magnivelare*. The second (B) and the third (C) major groups are separated at an ED of 9.63. Nine species with little CS integrate "B" group. The "C" group has three very particular species with variable CS (*Gomphus clavatus*, *Sparassis crispa *and *T. magnivelare*). The closest species were *Hypomyces lactifluorum *and *Hydnum repandum s.l *(ED = 1.40); *Lactarius volemus s.l*. joins these two species at a ED of 1.67. Other pairs of close species were *Laccaria laccata *var. *pallidifolia *and *Hygrophorus russula*, *Laccaria vinaceobrunnea s.l*. and *Cortinarius *secc. *Malacii *sp., *Lactarius deliciosus s.l*. and *Cantharellus cinnabarinus*, *C. cibarius *sp.1 and sp.2, *Ramaria *spp. and the *A. caesarea *complex. There were also three minor groups of species, *H. lactifluorum*,* H. repandum s.l*., *L. volemus s.l.*, *Agaricus pampeanus *and *N. lepideus *which were clustered as group "d" at a ED of 4.53. *Lactarius deliciosus s.l.*, *C. cinnabarinus *and *Austroboletus betula *conformed group "e", which was joined at a ED of 5.78. *Laccaria laccata *var. *pallidifolia*, *H. russula*, *Hygrophoropsis aurantiaca*, *L. vinaceobrunnea s.l*. and *Cortinarius *secc. *Malacii *sp. integrated group "f", which couples at a ED of 6.21.

**Figure 8:**
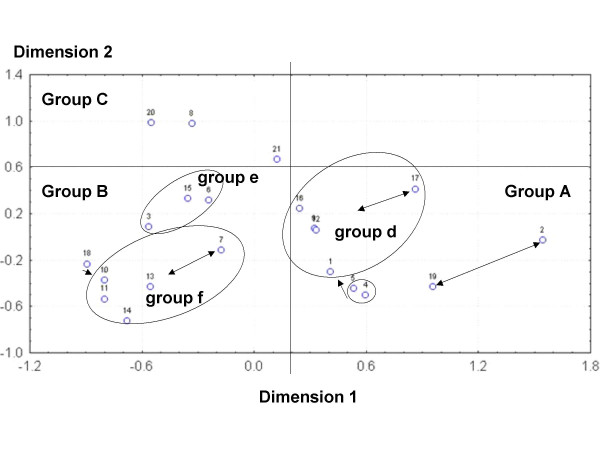
**Multi-dimensional scaling of fungal species by their euclidean distances**. Inside lines divide mayor mushroom groups designed by capital letters. Ellipses show minor mushroom groups designed by lowercase letters. Arrows show differences with tree diagram groups. 1: *Agaricus pampeanus*; 2: *Amanita caesarea *complex; 3: *Austroboletus betula*; 4: *Cantharellus cibarius *sp.1; 5: *Cantharellus cibarius *sp.2; 6: *Cantharellus cinnabarinus*; 7: *Cortinarius *secc. *Malacii *sp.; 8: *Gomphus clavatus*; 9: *Hydnum repandum s.l.*; 10: *Hygrophoropsis aurantiaca*; 11: *Hygrophorus russula s.l.*; 12: *Hypomyces lactifluorum*; 13: *Laccaria vinaceobrunnea s.l.*; 14: *Laccaria laccata *var.* pallidifolia*; 15: *Lactarius deliciosus s.l.*; 16: *Lactarius volemus s.l.*; 17: *Neolentinus lepideus*; 18: *Pleurotus *sp.; 19: *Ramaria *spp.; 20: *Sparassis crispa*; 21: *Tricholoma magnivelare*.

**Figure 9:**
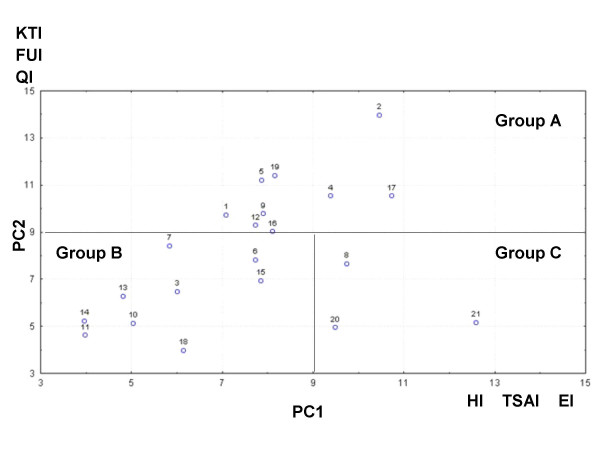
**Principal component analysis of edible mushrooms, obtained from cultural significance sub indexes**. Inside lines divide mayor mushroom groups designed by capital letters. PC: Principal component; EI: Economic Index; FUI: Frequency of Use Index; HI: Health Index; KTI: Knowledge Transmission Index; QI: Mention Index; TSAI: Taste Score Appreciation Index. 1: *Agaricus pampeanus*; 2: *Amanita caesarea *complex; 3: *Austroboletus betula*; 4: *Cantharellus cibarius *sp.1; 5: *Cantharellus cibarius *sp.2; 6: *Cantharellus cinnabarinus*; 7: *Cortinarius *secc. *Malacii *sp.; 8: *Gomphus clavatus*; 9: *Hydnum repandum s.l.*; 10: *Hygrophoropsis aurantiaca*; 11: *Hygrophorus russula s.l.*; 12: *Hypomyces lactifluorum*; 13: *Laccaria vinaceobrunnea s.l.*; 14: *Laccaria laccata *var. *pallidifolia*; 15: *Lactarius deliciosus s.l.*; 16: *Lactarius volemus s.l.*; 17: *Neolentinus lepideus*; 18: *Pleurotus *sp.; 19: *Ramaria *spp.; 20: *Sparassis crispa*; 21: *Tricholoma magnivelare*.

The MDS analysis two-dimensional solution (stress = .00048) is displayed in Figure 8. The species configuration was similar to the grouping in the cluster technique. Some species pairs disappeared like *Ramaria *spp.-*A. caesarea *complex and *L. vinaceobrunnea s.l.*-*Cortinarius *secc. *Malacii *sp. In minor group "d", *N. lepideus *was far away from the rest of the species and the two *C. cibarius *species were closely related to this group. *Cortinarius *secc. *Malacii *sp. was far away from the rest of "f" species and *Pleurotus *sp. joined them. The two first axes separated quite well between the major groups. Species in group "A" always had values over 0.2 in dimension 1, species in group "B" always had values under 0.2 in dimension 1 and lesser than 0.6 in dimension 2, while "C" group species were always over 0.6 in dimension 2 (Figure 8). These three major groups were also supported by the PCA (Figure 9). In it, the first three principal components (PC) explain cumulatively 71.67% of data variation. The most important variables in PC1 were the economic index, taste score appreciation index and health index (eigenvalues 0.52, 0.49, 0.46 respectively). In PC2, most important variables were the knowledge transmission index, frequency of use index and mention index (eigenvalues 0.50, 0.49, 0.44 respectively). Then, the species on group "A" (nine of the most culturally significant) in general are characterized by: have being used by more than three generations, are consumed frequently (except *Neolentinus lepideus*) and because they are the best known species; they also have some economic importance (particularly *N. lepideus*, the *Amanita caesarea *complex and *Cantharellus cibarius *sp.1), pleasant tastes, and are considered health enhancing (except the *A. caesarea *complex). The species on group "B" have zero economic importance, have mediocre to appreciable tastes, are not traditionally relevant (except *Cortinarius *secc. *Malacii *sp. and *Laccaria vinaceobrunnea s.l.*), are consumed infrequently and are known by less than 50% of informants. The three species in "C" in general are not consumed traditionally in Ixtlan, are used infrequently and are known by few informants (except *Tricholoma magnivelare*); on the other hand, they have a combination of high economic importance, pleasant tastes and health promoting properties.

**Figure 10:**
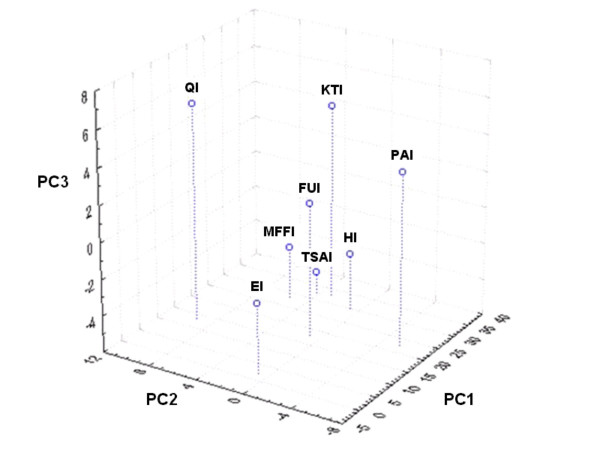
**Principal component analysis of cultural significance sub indexes from the species values**. PC: Principal component; EI: Economic Index; FUI: Frequency of Use Index; HI: Health Index; KTI: Knowledge Transmission Index; MFFI: Multifunctional Food Index; PAI: Perceived Abundance Index; QI: Mention Index; TSAI: Taste Score Appreciation Index.

In the PCA between sub indexes, the first three components explain 89.92% of data variation. There were not sub index groupings; just MFFI and TSAI were related. The HI was near these and the rest were isolated in the extremes (Figure 10).

The EI was the most correlated variable; it had positive correlations with the mention index, health index and taste score appreciation index (r_s _= 0.651, P = 001; r_s _= 0.638, P = 0.002; r_s _= 0.546, P = 0.011 respectively). People obtain edible mushrooms in Ixtlan easily; there are several acquisition mechanisms such as collect, reciprocal gifting and buying, thus almost every person have access to this resource [24]. Then, these correlations are logic because the prices of mushrooms are low with the exception of those having very good tastes or considered as "specials", "like meat" "very consistent" or with health enhancing properties. Sold species had high mentions because people knowing just few species always mentioned them [46]. There was also a correlation between the multifunctional food index and the taste score appreciation index (r_s _= 0.451, P = 0.040). Since mushrooms tasting better are coked in ways to conserve their flavor and thus they are not mixed with many other elements.

The rest of the possible correlations (24/28) were not significant, this and data from PCA between sub indexes, are relevant because they showed that different cultural domains evaluated behaved in different directions and, with some exceptions, were independent. Methodologically this must be expected because a cultural domain is defined as "... an organized set of words, concepts or sentences, all of the same level of contrast, that jointly refer to a single conceptual sphere" [47] thus our variables were defined accurately and did not represented unbound elements that could cause confusion between interviewees.

### Implications for further ethnobiological studies

The model proposed here is based on the fact that CS of resources is determined by a wide number of variables or cultural domains. To propose an index that includes all possible cultural domains is not feasible since cultures around the world value different resources attributes; its better to develop indexes composed by cultural domains locally important and relevant to the studied organisms. If a core of main cultural domains is used, some of them can be added or removed and the opportunity of cross-cultural analysis remains. This is possible by contrasting the relative positions of species in several compound indexes (also possible for individual variables) using rank correlations. Rank correlations allow the comparison of the species among cultural significance gradients and are not affected by scale or methodological differences between studies. Some subjects that could be assessed with this approach are: the relative position of species within the whole CS estimates, the relative position of species within each cultural domain and the relative weight of each variable determining de CS of species in different cultural contexts. These data are necessary to answer why cultures use some resources from their surroundings and no others? Why different societies use different species even if they are exposed to similar environments? How does human societies structure their subsistence strategies in function of what they have and what they believe? In sum, to give a step forward to a more integrative and explicative ethnobiology.

Caution must be taken when using compound indexes; as they imply a question for every variable for each species known by an informant, wise informants have to deal with a huge number of questions. In our case knowledgeable ones answered 7 questions by 21 species; that was a questionnaire of 147 questions that toke at least 3–4 hours. This spent much time and disposition from the interviewee or two or three sessions to complete the task. Furthermore, if more variables, more species or bigger informant samples are to be included, serious logistic efforts as long field journeys, enough resources and interviewees time must be considered.

## Conclusion

The mushrooms with highest cultural significance in Ixtlan according with the EMCSI were *C. cibarius s.l*. and the *A. caesarea *complex. Other studies have demonstrated that these species have high cultural significance in other places in Mexico, even in the world. However to understand if this is a common pattern still remains uncertain and waiting for wider geographical studies.

In each sub index the species had different behaviors; still in specific universes, as edible mushrooms, people value species for many different reasons. This has enormous implications because it tells that cultural significance of organisms is a complex construction with multifactor causes.

The multivariate analysis showed hidden patterns such as species groupings and the conjunct of variables determining such arrangement; so the compound index is a powerful tool to understand the causes of the cultural significance of resources.

The low degree of correlation among our variables showed that each is an independent process influencing the cultural significance of edible mushrooms. Correlations also highlighted that the economy is a factor that could influence several other variables, thus it has to be analyzed with more detail in areas where the commercialization of resources is important.

The relative positions of species within the cultural significance gradient are more useful than their absolute values on the index, because they bring the opportunity of cross-cultural analysis if rank correlations are used to compare several species listings. This approach can also be used in intracultural studies because it is flexible and allows including or removing cultural variables to adjust it to the nature of the cultures or resources under study.

However, the method proposed have some logistical limitations, it need big samples and large questionnaires, thus it is very time consuming. An alternative for this could be to ask informants just a random sample of species from their free listings. The compound index also needs a previous and profound background of the traditional local knowledge. This can be saved performing a previous inquiry, by the interviewee's point of view, about the cultural domains locally relevant.

## Abbreviation

CFSI: Pieroni's Cultural Food Significance Index

CS: Cultural significance

ED: Euclidean distance

EI: Economic Index

EMCSI: Edible Mushrooms Cultural Significance Index

FUI: Frequency of Use Index

HI: Health Index

ICS: Turner's Index of Cultural Significance

KTI: Knowledge Transmission Index

MDS: Multi-Dimensional Scaling

MFFI: Multifunctional Food Index

PAI: Perceived Abundance Index

PC: Principal Component

PCA: Principal Component Analysis

QI: Mention Index

TSAI: Taste Score Appreciation Index

UV: Use value

## Authors' contributions

RG-O designed the study, carried out the field work, the analysis and interpretation of data and wrote the manuscript. JC, AE-T and JC participated in the design of the study, and made substantial contributions to the analysis and the revision of the document. All authors read and approved the final manuscript.
